# Lumbar Disc Herniation: Comparing Pain Relief After Medical and Surgical Intervention

**DOI:** 10.7759/cureus.15885

**Published:** 2021-06-24

**Authors:** FNU Abdullah, Amerta Bai, FNU Sahil, Deepak Kataria, Mohammed Abbas, Farhan Ullah, Sidra Naz, Amna Jamil, Aliya Fatima, Sidra Memon

**Affiliations:** 1 Neurology, Jinnah Sindh Medical University, Karachi, PAK; 2 Internal Medicine, Shaheed Mohtarma Benazir Bhutto Medical University, Larkana, PAK; 3 Neurology, Shaheed Mohtarma Benazir Bhutto Medical University, Larkana, PAK; 4 Internal Medicine, University of Louisville, Louisville, USA; 5 Internal Medicine, Ayub Teaching Hospital, Karachi, PAK; 6 Internal Medicine, University of Health Sciences, Lahore, PAK; 7 Internal Medicine, Jinnah Postgraduate Medical Centre, Karachi, PAK; 8 Internal Medicine, Jinnah Sindh Medical University, Karachi, PAK

**Keywords:** lumbar disc herniation, lumbar discectomy, medical management, pain management, degenerative lumbar disorders

## Abstract

Introduction

The most common degenerative abnormality of the lumbar spine is lumbar disc herniation. There are two options of treatment, i.e. medical and surgical. Due to the scarcity of literature, it is a need of the hour to further study and evaluate the benefits and efficacy of early surgical intervention versus conservative management of lumbar disc herniation.

Methods

This study was conducted in the neurology unit of a tertiary care hospital in Pakistan from April 2019 to March 2021. After obtaining informed consent, 250 patients with a lumbar disc herniation, between the ages of 20 and 50 years, were enrolled in the study. Out of them, 81 participants chose surgical intervention while 169 participants chose medical intervention. Before the intervention, the patient’s pain score was noted on the visual analog scale (VAS). The pain was assessed again 14 days after surgical intervention and 90 days after the start of medical intervention.

Results

There was a significant difference in the pain score in the post-intervention period in both the medical (7.01 ± 1.05 vs. 3.54 ± 0.51; p-value: <0.0001) and surgical intervention groups (6.92 ± 0.95 vs. 2.41 ± 0.42; p-value: <0.0001). Post-intervention, the VAS pain score was significantly lower in the surgical group as compared to the medical group (2.41 ± 0.42 vs. 3.54 ± 0.51; p-value: <0.0001).

Conclusion

In this study, there was a significant decline in pain in both groups; however, the reduction was more significant in the surgical group. Patients should be given both options for management of lumbar disc herniation and should be explained the pros and cons of each treatment option.

## Introduction

The most common degenerative abnormality of the lumbar spine is lumbar disc herniation. It is prevalent in 2%-3% of the world’s population and mainly affects people in their fourth and fifth decades of life. It presents clinically as low back pain, followed by progressive sciatica. Its causes are multifactorial, including mechanical and genetic components. Smoking and repetitive mechanical loads and vibration play a major role as well [1]. It is usually diagnosed clinically while magnetic resonance imaging (MRI) is the diagnostic imaging of choice for lumbar disc herniation [2-3]. The management usually involves patient education, physical therapy, pharmacotherapy, and surgical intervention [4].

The objective of treatment is to relieve pain and help in neurological recovery with early return to daily work. Young patients with small disc herniation and mild neurological deficits are mainly treated conservatively [5]. Many advancements have been made in management recently. According to the recent clinical trials, it has been observed that recovery rate, as measured by the Japanese Orthopedic Association, is faster in the group with early surgery and favors an early return to work [6]. However, another research concludes that recovery by surgery takes longer, i.e. 11 weeks, whereas conservative treatment requires recovery in seven weeks [4].

Although a considerable amount of work has already been done globally, there is very limited data available in the local setting. Therefore, it is the need of the hour to further study and evaluate the benefits and efficacy of early surgical intervention versus conservative management of lumbar disc herniation.

## Materials and methods

This study was conducted in the neurology unit of a tertiary care hospital in Pakistan from April 2019 to March 2021. After obtaining informed consent, 250 patients with lumbar disc herniation of either gender, between the ages of 20 to 50 years, were enrolled in the study via consecutive convenient non-probability sampling. Lumbar disc herniation was confirmed by an MRI scan. Ethical review board approval was taken before the enrollment of participants.

After enrollment, patients’ demographics and disc herniation location were noted in a self-structured questionnaire. Patients were counseled about the advantages and disadvantages of both medical and surgical treatment. Based on their preference, patients were enrolled in either the medical or the surgical intervention group. Surgical intervention was chosen by 81 participants while 169 participants chose medical intervention. Before the intervention, the patient’s pain score was noted on the visual analog scale (VAS). The VAS has markings from zero to 10, where the former represents no pain and the latter represents worse pain.

Surgical intervention was done via minimally invasive lumbar discectomy 10 days after the enrollment of patients. The medical intervention included 75 mg pregabalin thrice daily and painkillers, i.e. paracetamol 450 mg and orphenadrine citrate 35 mg, each twice daily. The pain was assessed again 14 days after surgical intervention and 90 days after the start of medical intervention.

During the study, 11 participants requested surgical intervention and were excluded from the study; 24 patients were lost to follow-up, three in the surgical intervention group and 21 in the medical intervention group. More people were lost to follow-up in the medical intervention group, due to the longer period of follow-up. All participants that completed the follow-up were included in the final analysis.

Data were processed and analyzed using the Statistical Packages for Social Sciences (SPAA) version 23.0 (IBM Corp., Armonk, NY). The mean and standard deviation (SD) were calculated for continuous variables; frequencies and percentages were calculated for categorical variables. The mean pain score was compared using the t-test. A p-value of less than 0.05 meant that there is a difference between the two groups and the null hypothesis was void.

## Results

No significant difference was found in the demographics and location of disc herniation between the medical and surgical intervention groups (Table 1).

**Table 1:** Comparison of demographics and location of disc herniation BMI: body mass index

Demographics	Medical intervention (n=137)	Surgical intervention (n=78)	p-value
Age in years (Mean ± SD)	39 ± 6	40 ± 6	NS
Male	70 (51.0%)	39 (50.0%)	NS
BMI more than 25 kg/m^2 ^	38 (27.7%)	23 (29.4%)	NS
Location of lumbar disc herniation			
L4-L5	101 (73.7%)	57 (73.0%)	NS
L5-S1	36 (26.3%)	21 (27.0%)	

There was a significant difference in the VAS pain score in post-intervention period in both medical (7.01 ± 1.05 vs. 3.54 ± 0.51; p-value: <0.0001) and surgical intervention group (6.92 ± 0.95 vs. 2.41 ± 0.42; p-value: <0.0001). Post-intervention, the VAS pain score was significantly lower in the surgical as compared to the medical group (2.41 ± 0.42 vs. 3.54 ± 0.51; p-value: < 0.0001) (Table 2).

**Table 2:** Comparison of pre-and post-intervention VAS score in both groups *calculated by comparing pre and post-intervention VAS score within the same group **calculated by comparing the post-intervention score of both groups VAS: visual analog scale

Group	VAS Score (Mean ± SD)		p-value	
	Pre-intervention	Post-intervention	Intragroup*	Intergroup**
Medical	7.01 ± 1.05	3.54 ± 0.51	< 0.0001	< 0.0001
Surgical	6.92 ± 0.95	2.41 ± 0.42	< 0.0001	

## Discussion

The results of our study demonstrated that there was a significant difference in VAS pain score in the post-intervention period in both the medical and surgical groups. Moreover, the post-intervention VAS pain score was found to be significantly lower in the surgical group as compared to the medical group. The results of our study are in line with other studies. In a randomized controlled trial (RCT), early surgery for sciatica that had lasted for six to 12 weeks was compared with prolonged conservative care for six months. The trial showed rapid pain relief after early surgery in comparison to prolonged conservative care [7]. Similarly, Nygaard et al. and Ng et al. also highlighted that delayed surgery after eight to 12 months of sciatica showed worse results than earlier surgery [8-9]. Moreover, some of the high-quality observational cohort series showed significantly worse results after prolonged conservative care compared with surgery [10].

One of the major advantages in previous studies of early surgery for patients is faster relief of leg pain, reassurance, and earlier return to daily activities of life. However, these advantages of surgery by six months follow-up and even at eight weeks were no longer significant among them. Most of the patients also faced discomfort because of delayed surgery, whereas up to 56% of the patients recovered without any surgery [11]. In previous studies, it was found that better symptoms, functional status, and reassurance were associated with surgical treatment but no significance on disability or work outcomes was observed at five years. However, disability or work outcomes, combined with many factors, are more likely related to medical treatment. These factors may include accommodations in the workplace, tasks, autonomy, satisfaction, other sources of income, local economic factors, etc. [12]. This indicated that patients who were treated surgically showed greater relief from symptoms and improvement in the functional status upon follow-up as compared with patients who had non-surgical treatment [13]. Most of the patients prefer conservative treatment due to the lower risk of complications but rapid pain relief is more associated with surgical treatment. Previous observational studies have also found rapid back pain reduction with surgical treatment [14-15]. Previous RCTs have also demonstrated rapid pain reduction in patients who received surgery, but no significant benefit of surgery over conservative treatment at long-term assessments of neurogenic symptoms was observed [7,16].

The study has its limitations. First, since it was conducted in a single institute, care should be taken while inferring the result to a larger group and the sample size may have not been very diverse. Second, only pain was measured as an outcome of medical and surgical intervention.

## Conclusions

In this study, there was a significant reduction in pain in both groups; however, the reduction was more significant in the surgical group. Patients should be given both options for the management of lumbar disc herniation and should be explained the pros and cons of each treatment option. Further large-scale studies are needed to assess the long-term impact of surgical and medical intervention.
